# “An uncomfortable topic”: Health professionals' perspectives on child protection capacities, training offers and the potential need for action in Germany

**DOI:** 10.1186/s12913-022-07905-7

**Published:** 2022-04-28

**Authors:** Anna Maier, Jörg M. Fegert, Ulrike Hoffmann

**Affiliations:** Ulm University Hospital, Department of Child and Adolescent Psychiatry/Psychotherapy, Steinhövelstr. 5, Ulm, 89075 Germany

**Keywords:** Continuing medical education, Health inequalities, Child maltreatment, Health professionals, Health Service, Prevention

## Abstract

**Background:**

Child maltreatment, due to its high prevalence and often long-lasting (health and/or psycho-social) consequences, is one of the main reasons for global health inequalities. The medical field offers many opportunities to support affected children. This gives physicians and other health professionals the opportunity to provide protective measures and therapies to affected children at an early stage. However, the level of training concerning child protection is often too low among health professionals. This can affect the quality of care as well as providing the appropriate treatment and thus, the long-term (health) burden. The present work aims to survey the state of knowledge and capacities of health professionals regarding child protection in medicine and elicit health professionals' perspectives who absolved a child protection online course on a potential need for action in Germany.

**Methods:**

From June 2016 until February 2021, 3,360 health professionals were interviewed. Using quantitative and qualitative items, the questionnaire assessed demographic and professional background information as well as assessments regarding the awareness of child protection, abilities in child protection among health professionals and training offers in medicine.

**Results:**

The analysis indicates that the topic of child protection in medicine is not as present as the high prevalence of child maltreatment would imply. The majority (94.0%; *n* = 3.159) of the health professionals stated that they need more knowledge and capacities regarding child protection in medicine. More than half of the health professionals assessed the importance of the issue of child protection as low among health professionals. The reasons cited included child protection as an uncomfortable topic, an unwillingness among managers, and a lack of training on the topic.

**Conclusions:**

There is too little awareness and importance regarding child protection in the medical field in Germany. Hence, it is difficult to ensure adequate care for those affected. Child protection topics should be mandatory in the training curricula of all health professionals, and quality standards for prevention and intervention should be implemented in medical institutions. Furthermore, networking in child protection has to be improved, and medical campaigns should address the topic to sensitize health professionals and society to the issue and to destigmatize the topic.

## Background

The World Health Organization (WHO) estimates that child maltreatment is one of the main causes of social and health inequalities worldwide [1]. Although there is no typical maltreatment syndrome as a result of maltreatment in childhood and adolescence, affected individuals may experience an increased risk for short- and long-term (psychological, somatic & social) consequences [2–4]. According to the results of international studies, 8% to 40% of children and adolescents affected by child maltreatment develop mental disorders [5, 6]. Frequently, impairments in memory, cognitive performance and cognitive control also occur in the presence of child maltreatment [7, 8]. In addition, the likelihood of a more unhealthy lifestyle and violent behavior is increased [2, 9–11]. The link between child maltreatment and numerous (mental) health sequelae and their lifelong impact on victims have been repeatedly confirmed [12].

The literature shows that physicians are among the privileged first contact persons for those affected by child maltreatment [13, 14]. Furthermore, physicians are in a very suitable position to recognize child abuse [15]. This gives physicians and other health professionals the opportunity to provide appropriate protective measures and therapies to those affected at an early stage and thus improve the (mental) health of children and adolescents. However, it is not only the task of physicians in the fields of pediatrics, child and adolescent psychiatry/psychotherapy or forensic medicine but also of all physicians and other health professionals, since child maltreatment cases can occur in all medical fields [16].

For health professionals in Germany, the Federal Child Protection Act currently regulates the procedure to follow in the event of suspected child maltreatment. Thus, if health professionals become aware of significant indications of child maltreatment in the course of their professional activities, they may first discuss the situation with the child and the legal guardians and, if necessary, urge the legal guardians to seek help. In addition, health professionals have a right to anonymous consultation with a child protection specialist. If the risk to the child cannot be averted together with the legal guardians, the Youth Welfare Office must be called to avert further danger for the child [17, 18].

However, it became clear that the level of training in the field of child protection in medicine is often too low among health professionals, and the WHO estimates that 90% of child maltreatment cases remain undetected by institutions [1, 19, 20]. This can affect the quality of care as well as the appropriate treatment and thus the long-term (health) burden on those affected.

Unfortunately, it has become apparent that there is very little evidence on the perspectives and experiences of health professionals regarding the state of training and quality standards in child protection and the needs and requirements of health professionals. This study collected quantitative and qualitative data on this topic to address the current gap in the literature by examining the views and experiences of health professionals who have completed continuing education on child protection.

This research aimed to analyze and reflect on the perspectives and experiences of health professionals who absolved a child protection online course to identify needs, barriers and opportunities in medical child protection among health professionals and to improve the treatment and management of children and adolescents affected by child maltreatment in the future.

## Methods

### Research design

From June 2016 until February 2021, the Department of Child and Adolescent Psychiatry/Psychotherapy at the University Hospital of Ulm in Germany offered a free-of-charge online course on child protection in medicine funded by the German Ministry of Health, which imparted knowledge and skills on epidemiology, risk factors, anamnesis, procedures and legal basis in cases of (suspected) child abuse [19]. The participating health professionals had to fill out questionnaires to evaluate the current training situation in medical child protection. There was a baseline survey before course-start and a post evaluation after absolving the online course successfully.

Altogether, 3,998 participants absolved the online course successfully and took part in the evaluation. Of those, 3,360 participants were included in the analysis, based on their profession as physicians, health care professionals, psychotherapists (in training), other therapists (e.g., occupational or speech therapists) (in training) or medical students. All data evaluated in this paper were collected in the evaluation of the online course. The evaluation was conducted in German.

Data include demographic and professional background information, the level of motivation for further training in child protection as well as assessments regarding the meaning of child protection, level of knowledge, capacities and self-efficacy in child protection among other health professionals and themselves.

The hypotheses behind this study stated that health professionals need more support and capacities in child protection and that targeted interventions can support them to do so. To prove these hypotheses, quantitative and qualitative research were performed. This design analyzed the capacities and needs of health professionals on the one hand and assessed their specific and individual needs on the other hand. The study is approved by the Ethics Committee of the medical faculty at the University of Ulm on June 16, 2016.

### Quantitative Data

**Fig. 1:**
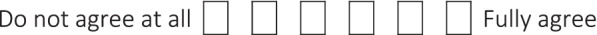
Endpoint-named scale to survey quantitative data

Demographic data, except age and professional experience, and assessments of the availability and status of training on child protection in medicine were collected using nominal and ordinal items, respectively. Quantitative data on personal experience, requirements of the participants concerning child protection, the self-assessment of knowledge, acting and emotional capacity as well as self-efficacy were measured by the agreement to statements using endpoint-named scales. So these data can be considered as interval scaled (Fig. 1) ([21], pp. 77). To better illustrate the results, the data on personal experience and assessment of the importance of child protection in medicine were categorized for Figs. 3 and 4.

Knowledge and emotional capacity were assessed using eight resp. six items related to the topic, and self-efficacy was assessed using the Schwarzer and Jerusalem General Self-Efficacy Scale [22].

For the sample description, data on personal experience and requirements descriptive analysis were calculated. Mean and standard deviation were calculated for metric and percentages for nominal and ordinal variables. To analyze the correlation between professional experience and the experience in child protection the Pearson correlation and a Kruskal–Wallis and subsequent post-hoc tests were calculated.

The descriptive and inductive statistics from the quantitative data were calculated using SPSS statistical software (Version 26) [23]. The figures were created with Microsoft Office 2019.

### Qualitative Data

The qualitative data were assessed by two open questions concerning the participants’ awareness of the topic of child protection in medicine. Both questions were only asked if attention to the topic of child protection in medicine was considered insufficient by the participant.

“What do you think are the reasons for insufficient awareness of the topic of child protection in medicine?”

“What do you think would be necessary for more attention to be given to the issue of child protection in medicine?”

The answers were studied in detail, and a qualitative content analysis according to Mayring was performed. This analysis combined quantitative analysis steps, such as categorizing the material and counting these categories, and qualitative ones, such as analyzing the statements. In the course of this analysis, inductive categories, which derive directly from the material without pursuing already existing presuppositions, were formulated step by step. Mayring hereby differentiates between four steps that are based on each other. In the first step, the research team discussed and defined the research questions and hypotheses (see section on Research design). Subsequently, an overview of the collected qualitative data was created by analyzing the qualitative data and dissecting it according to a previously defined procedure in order to ensure intersubjective comprehensibility. In the course of this, all individual statements were extracted from the participants' statements and documented individually. Based on this, codes were created from the data by extracting the content of each individual statement made by the participants. The coding and categorization were discussed and defined within the research team. To ensure the objectivity of the analysis, independent raters also analyzed the qualitative data and created categories. These categories were then compared with the categories already formed and adjustments were made where necessary [24].

## Results

### Sample Description

**Fig. 2:**
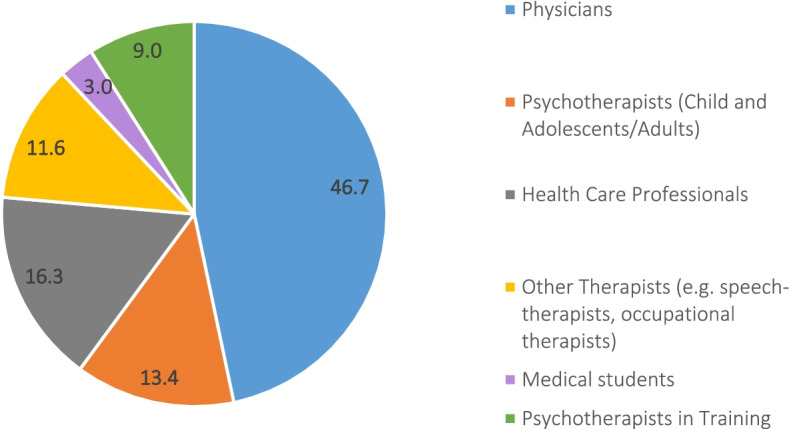
Distribution of professional groups within the study sample in % (*N* = 3,360)

The 3,360 health professionals who were analyzed in the study were 39.5 years old on average (standard deviation (Stdv) = 10.5). A total of 80.9% (*n* = 2,718) of the health professionals were employed, 16.5% (*n* = 553) were self-employed and 6.3% (*n* = 211) were unemployed or on parental leave. Figure 2 shows the distribution of the health professionals in the different professional groups.

Table 1 presents the different institutions in which the health professionals were employed. Therefore, most of the analyzed health professionals worked in a clinical context, especially in the departments of Child and Adolescent Psychiatry/Psychotherapy or Pediatrics and Adolescent Medicine.

**Table 1:** Institutions that employed the health professionals (*N* = 3,360)

Institution (multiple replies possible)	*n*	% of 3,360
Department of Child and Adolescent Medicine	991	29.5
Department of Child and Adolescent Psychiatry/Psychotherapy	477	14.2
Other Practice	324	9.6
Other Clinic Department	253	7.5
Public Health Services	223	6.6
Practice of Child and Adolescent Medicine	218	6.5
Practice of Child and Adolescent Psychiatry/Psychotherapy	128	3.8
Youth Services	128	3.8
Practice of Psychiatry/Psychotherapy (Adults)	99	2.9
Department of Psychiatry/Psychotherapy (Adults)	77	2.3
Department of Psychosomatics	62	1.8
Ambulatory nursing and pediatric care	39	1.2
Self-employed Midwife	37	1.1

**Fig. 3:**
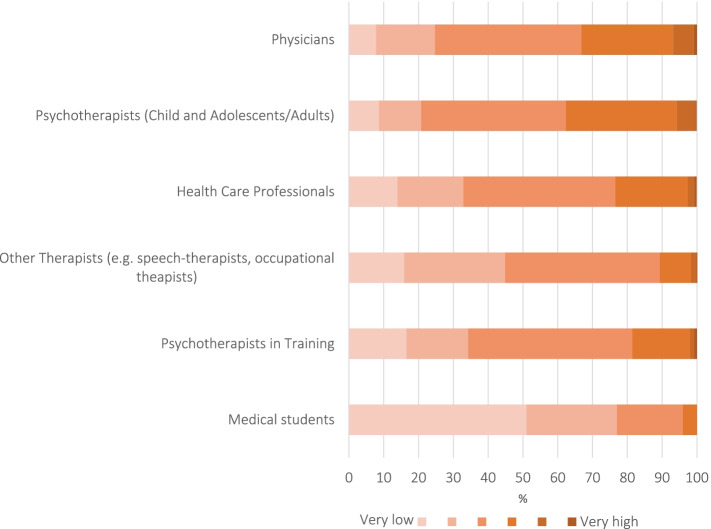
Participants' self-assessed professional experiences with child protection in medicine (*N* = 3,360)

The health professionals gained professional experience for an average of 10.9 years (Stdv: 9.7); nevertheless, they estimated their personal experiences in child protection in the lower area (Fig. 3).

### Self-assessment of capacities in child protection

The level of the participants’ self-assessed professional experiences in Fig. 3 indicates that the topic of child protection in medicine is not as present as the high prevalence of child maltreatment would imply and underlines the WHO’s estimation that 90 % of child maltreatment cases remain undetected by institutions. Therefore, it is difficult to ensure adequate care for those affected. The majority (94.0 %; *n* = 3,159) of the health professionals included in this study already recognized this problem and stated that they need more knowledge and capacities in child protection in medicine. Table 2 outlines the level of self-estimated knowledge and capacities in child protection between the different types of health professionals. Here, a distinction was made according to professional group and level of training in order to show the level of knowledge and capacities in the various areas of the healthcare sector in detail.

**Table 2:** Self-assessment of knowledge and capacities in child protection in medicine on average (*N* = 3,360)

	Mean (Stdv)					
**Knowledge**^1, *^	26.1 (6.5)	28.2 (6.7)	23.9 (7.2)	21.8 (6.9)	20.6 (6.7)	26.5 (6.8)
**Acting Capacity**^**2**^	3.4(0.9)	3.5(0.9)	3.1(0.9)	2.8(0.9)	2.5(0.9)	3.3(0.9)
**Emotional Capacity**^3, *^	26.8(3.8)	28.2(3.7)	26.7(4.1)	26.6(4.4)	24.3(4.7)	27.5(3.9)
**Self-efficacy**^4, *^	24.3(5.1)	26.6(4.3)	23.9(5.4)	23.5(5.5)	22.0(6.6)	25.6(4.7)
	**Physicians**	**Psychotherapists** (Child and Adolescents/Adults)	**Health Care Professionals**	**Other Therapists** (e.g., speech-therapists, occupational therapists)	**Medical students**	**Psychotherapists in training**

^1^Min: 8; Max: 48, ^2^ Min: 1; Max: 6, ^3^ Min: 6; Max: 36, ^4^ Min: 4; Max: 40, ^*^These parameters were surveyed by a score

Table 2 shows that health professionals estimate their own knowledge and capacities in child protection in the upper middle field. Nevertheless, psychotherapists in training, other therapists and health care professionals, and especially medical students, estimate their own knowledge and capacities as rather low.

It seems that even before training in child protection, the participants estimated their own level of knowledge and capacities in child protection to be higher than the average knowledge and capacities in their professional group. This was also confirmed by a significant Pearson correlation between professional experience in years and the self-assessment of experience in child protection (r = 0.309; *p* < 0.001; *n* = 3,360). Moreover, a Kruskal–Wallis and subsequent post-hoc tests revealed only weak differences between the professional groups according to Cohen's d.

This shows the high importance of reaching and training health professionals who have a lower level of knowledge and capacities in child protection and/or are still in training as well as obviously are not highly represented in the study sample. For this reason, we surveyed the assessment of training offers and services in the field of child protection in medicine.

### Assessment of training offers and services

It became clear that most of the health professionals (*n* = 2,639; 78.5%) did not estimate the training offers and services in child protection as sufficient; however, almost all of them (*n* = 3,317; 98.7%) indicated a high need for a larger dissemination of knowledge and abilities in child protection among health professionals. Moreover, broader and more flexible access to training offers and services is required.

The majority of the health professionals (*n* = 3,159; 94.0%) mentioned that they needed more knowledge and capacities in child protection in their daily professional routines, and for almost half of them (*n* = 1,668; 49.6%), the offered online course was the only training offer available.

### Importance of child protection in medicine

**Fig. 4:**
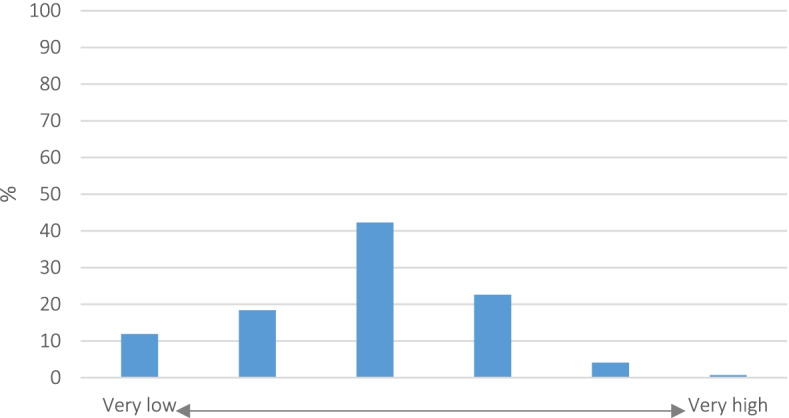
Assessment of the importance of child protection in medicine (%) (*N* = 3,360)

Figure 4 shows that more than half of the health professionals assessed the importance of the issue of child protection as being rather low in the medical field.

As reasons for this, the health professionals especially mentioned a lack of knowledge and time as well as fears of coming into contact with a case of child maltreatment.

To find the underlying cause of these issues, the qualitative statements of the health professionals were analyzed concerning the reasons for the low importance of child protection and resulting needs in the medical field. Four key themes could be identified.

#### Awareness in society, politics and the media

The participants pointed out that, in their opinion, the topic of child protection should receive more importance in society and politics and that measures of a structural or financial nature, for example, should be implemented on this basis. Currently, the importance is still low because child abuse is an *"inconvenient topic" (quote from an interviewed health professional).*

The interviewees also emphasized the role of the media, stating that child maltreatment, its origins and consequences should be more strongly addressed in the media, rather than simply scandalizing cases of maltreatment, and should be included in campaigns to sensitize society as a whole to the issue and to destigmatize the topic.

#### Continuing medical education, especially for nonspecialist health professionals

Numerous respondents commented that more knowledge and competencies, and thus more training, of health professionals on the topic of child protection is necessary. 

*"More awareness-raising activities among all staff groups and specific training opportunities."*

*(quote from an interviewed health professional)*

In addition, nonmedical or nonspecialist staff, in particular, would need training on the topic of child protection, as they may also come into contact with cases of child maltreatment. There was also a call for better support in child protection cases. 

*"Low-threshold offers for physicians in case of questions, advice in the event of suspicious cases, also availability after hours or in the evening, support regarding local contact addresses." *

*(quote from an interviewed health professional) *

Another point of criticism concerned the education and training of health professionals, which often does not address the topic of child protection. This gap also needs to be closed.

#### Support in child protection cases by executive

Some of the respondents would like to have more support from managers in their institutions in the context of child protection cases. There are reports of managers who explicitly do not want to address the topic of child protection in their facility.

*"Courage to be vigilant for child maltreatment by those in authority."*

*(quote from an interviewed health professional)*

On the one hand, support in dealing with suspected cases would be important to the respondents, but on the other hand, support in further training on the topic would also be essential. Furthermore, there is a requirement for a child protection specialist for each medical facility.

#### Networking between all professionals involved

Dealing with child protection cases usually requires different professional groups in different institutions. In addition, it was noted that better networking between all professionals involved in child protection, within but especially beyond the medical field, is necessary and should therefore be established and financially supported in all medical institutions. This also includes the knowledge of the other professionals involved in cases of child maltreatment, their fields of activity and possibilities.

*"Establish networking with external resources for practices and clinics as a mandatory structural requirement."*

*(quote from an interviewed health professional)*

Some respondents already reported successful networking and its success in dealing with cases of child maltreatment.

## Discussion

The results of this work show that the participants in this study, who took part in an online course on child protection in medicine, rated the knowledge of child protection among health professionals as too low. The results allow for the conclusion that the level of knowledge among the surveyed professionals is, on average, higher than the level among all health professionals. Therefore, we assumed that the participants are particularly committed and sensitized to the topic of child maltreatment. Similar trends can be observed internationally, although the current state of research on this issue is not yet broad enough [25–28].

In the field of education and training, different areas become clear in which increased efforts on child protection should be realized: first, in the area of health professionals’ education. The study showed that health professionals who are currently in training consider their competencies in child protection to be rather low. Although there were no clear differences in the assessment of knowledge and competencies between the professional groups considered in the analysis, it became clear that the higher the level of professional experience, the higher the assessment of one's own competencies in child protection. Thus far, medical training does not address the topic sufficiently. This confirms the demand of the "German Society for Child Protection in Medicine", which calls for child protection to be part of the training of every physician working in child and adolescent medicine [29].

As child protection cases can appear in all areas of the health system, (non-) physician health professionals that do not work with children and adolescents on a daily basis, such as nurses or employees of a general practitioner's office, should also be trained on the topic. This also confirms the demand of Berthold and colleagues to implement the topic obligatory in the education and training of all health professionals. In addition, these recommendations suggest that medical facilities maintain a multiprofessional child protection group. This interdisciplinary approach could also be used to better train all physicians, not just those in traditional "pediatric specialties" such as dentists, who play for example a significant role in the field of dental neglect. [30]. These training offers should be as barrier-free and easily accessible as possible. The online course "Child protection in medicine—a basic course for all health professionals", whose participants were interviewed for this study, offers a good opportunity for this due to its flexible nature [19].

Finally, the interviewees in this study repeatedly emphasized that they did not feel sufficiently supported or where even hindered by their executives in child protection cases. However, executives play a decisive role in the establishment of child protection measures because the importance that a manager attaches to this topic will also determine how employees handle it. Child protection in institutions is a relevant topic for management staff since the issue of child maltreatment affects a wide range of institutions. At the same time, an emotional topic requires a high degree of sensitivity on the part of executives [31]. Training on child protection should therefore also include a special focus on education and raising awareness in management staff. It is imperative that management staff acquire knowledge and skills in the area of child protection and reduce fears of contact with child maltreatment. To this end, the Department of Child and Adolescent Psychiatry/Psychotherapy at Ulm University Hospital, together with the German Hospital Federation, offers the E-Learning “Basic knowledge of child protection in institutions – an online course for managing positions” free of charge [32].

The statements of the interviewed health professionals clearly show that there still seem to be many gaps in dealing with child protection cases, especially in the area of exchange and networking. Studies on networking in the field of child protection indicate that there is a coexistence rather than cooperation in child protection. In pedagogical and medical institutions, there is a clear divide with the youth welfare office due to the fear of losing the trust of affected children, which has a negative effect on the health professionals’ willingness to cooperate. The fact that youth welfare offices can provide hardly any feedback on the further course of cases after a report for reasons of data protection makes cooperation more difficult, especially in crises. Mutually divergent expectations of the roles and tasks of the cooperating institutions and unclear responsibilities are also problematic [33–35].

Effective and sustainable child protection, however, requires a functioning network between all professions and institutions involved. Easy communication and long-term cooperation often enable low-threshold solutions to problems. In principle, the exchange of information and networking in child protection should not only take place in a case-related manner during a crisis but should be established beforehand. In this context, it is important to understand the theoretical foundations and models of thought, as well as the possibilities for action and limits of all professionals involved in child protection, and to develop joint case definitions, risk assessments and standards and guidelines for action [36, 37].

In addition, quantitative and qualitative interviews with health professionals have shown that the media should give more attention to the topic of child protection. However, the aim is not to scandalize the topic and spread anxiety but to reduce the fear of contact with cases of maltreatment and to sensitize society to the topic. In addition, the stigmatization of the topic should be reduced. In Germany, there are a number of campaigns, such as "Those who break the silence break the power of the perpetrators", a campaign that used video spots on all major German TV stations in 2010 to encourage sexually abused persons to break their silence [38, 39]. Another example is the "Have Courage!" initiative, which aims to prevent sexual abuse of children and young people by strengthening and promoting educational initiatives. The initiative refers to the approach of the UN Convention on the Rights of the Child and a comprehensive concept of sexuality education. In this context, the "Have Courage!" initiative also offers materials and information for educational professionals [40]. Evaluation of the initiative suggests that it is effective in preventing child maltreatment by providing knowledge, self-protection skills and awareness [41]. The "No Room for Abuse" initiative, which aims to make all facilities and organizations in Germany that work with children and adolescents both a place of protection and a place of competence for children and adolescents with regard to sexual abuse, also addresses medical facilities and health professionals [42]. All of the abovementioned campaigns focus on sexual abuse, rather than physical or emotional abuse or neglect. However, the prevalence of these forms of maltreatment is also persistently high [43]. Internationally, for example, the “Florida Winds of Change” campaign was designed to raise public awareness of child abuse and neglect and has been positively evaluated [44]. A Canadian study also demonstrated an improvement in parents' understanding of causes of and assistance with child maltreatment through a media-based component of a broader prevention strategy [45]. Thus, international campaigns, while broader in scope, are still very rare. Furthermore, it appears that campaigns to raise awareness of child maltreatment among health professionals are still lacking, both for Germany and internationally. Evaluations of such campaigns may allow conclusions to be drawn on how society and professionals can be reached particularly well.

### Limitations

The present study is limited in terms of its transferability of findings to the full health sector, as it analyzed a nonrepresentative sample of health professionals who have a special interest in the field of child protection. Furthermore, it is important to note that questions may be answered differently in the context of a training evaluation than in a stand-alone survey on training in medical child protection. However, because the goal of the work was to identify individual perspectives and opportunities for improvement in child protection the validity of the data is given. As the sensitized target group was particularly important to the research design by having experience with and insights into the handling of child protection cases in medicine of which they were able to report. Indeed, the participants in this study can be considered experts in the field of child protection in medicine, as they are familiar with both the everyday medical practice and the topic of child protection. To establish comparability of the results with other countries, it would be necessary to use the same questions that were used in this study. It can be assumed that, at least in part, structural and cultural factors in Germany contributed to the results. The quotes from the interviewees presented to illustrate the research results were gathered in German, translation bias in the content of the statements was addressed through professional language editing. Regardless of these limitations, this participatory research leads to a better understanding of the needs of health professionals in the field of child protection.

## Conclusions

The results of this work indicate that increased training, networking and awareness are needed among society and professionals about child maltreatment and its health consequences.

A major problem here is a lack of time resources among professionals, as well as fear of contact with and stigma surrounding child maltreatment. A shortage of health professionals and funding attributes to the lack of time resources. Fear of contact with cases of maltreatment and stigmatization of those affected by it can lead to further (mental) health impairments among them [46, 47]. Rüsch and colleagues identified three strategies as ways to reduce stigma: "Protest", "Education" and "Contact". “Protest” addresses stigmatizing public statements, media reports, and advertisements to change their impact. “Education” seeks to reduce stigmatizing attitudes and stereotypes by providing knowledge through various formats. “Contact” with people with mental illness should help reduce stereotypes and stigma [47].

The results of this work on improving training, networking, and awareness in the media on the topic of child maltreatment reflect these strategies. This demonstrates, once again, the importance of these strategies and the need for action in administration, policy and society.

## Authors information

Dr. Anna Maier is a research associate in the working group "Knowledge Transfer, Dissemination, E-Learning" and supervises several projects in the field of e-learning and child protection. She conducts research on the transfer and dissemination of knowledge between science and practice as well as prevention in the field of child protection.

Prof. Jörg M. Fegert is the Medical Director of the Clinic for Child and Adolescent Psychiatry/Psychotherapy at Ulm University Hospital and Head of the Competence Centre Child Protection in Medicine Baden-Württemberg. In addition, he is a member of numerous scientific and political advisory committees and heads a wide range of research areas in the transition from research to practice in child protection. He has been researching the prevention of Child Sexual Abuse and clinical issues for 30 years.

Dr. Ulrike Hoffmann heads the working group "Knowledge Transfer, Dissemination, E-Learning" in the Department of Child and Adolescent Psychiatry/Psychotherapy at Ulm University Hospital. She was involved as a research assistant in several projects on the topic of child protection at the University Hospital Ulm. Her research focus is on protection concepts in medical institutions.

## Data Availability

The datasets generated and/or analyzed during the current study are not publicly available due to reasons of data security but are available from the corresponding author on reasonable request.

## Abbreviations

Stdv: Standard deviation
WHO: World Health Organization
